# Quality and Quantity of Information in Summary Basis of Decision Documents Issued by Health Canada

**DOI:** 10.1371/journal.pone.0092038

**Published:** 2014-03-20

**Authors:** Roojin Habibi, Joel Lexchin

**Affiliations:** 1 Faculty of Health Sciences, McMaster University, Hamilton, Ontario, Canada; 2 School of Health Policy and Management, York University, Toronto, Ontario, Canada; 3 University Health Network, Toronto, Ontario, Canada; 4 Department of Family and Community Medicine, University of Toronto, Toronto, Ontario, Canada; University of New South Wales, Australia

## Abstract

**Background:**

Health Canada’s Summary Basis of Decision (SBD) documents outline the clinical trial information that was considered in approving a new drug. We examined the ability of SBDs to inform clinician decision-making. We asked if SBDs answered three questions that clinicians might have prior to prescribing a new drug: 1) Do the characteristics of patients enrolled in trials match those of patients in their practice? 2) What are the details concerning the drug’s risks and benefits? 3) What are the basic characteristics of trials?

**Methods:**

14 items of clinical trial information were identified from all SBDs published on or before April 2012. Each item received a score of 2 (present), 1 (unclear) or 0 (absent). The unit of analysis was the individual SBD, and an overall SBD score was derived based on the sum of points for each item. Scores were expressed as a percentage of the maximum possible points, and then classified into five descriptive categories based on that score. Additionally, three overall ‘component’ scores were tallied for each SBD: “patient characteristics”, “benefit/risk information” and “basic trial characteristics”.

**Results:**

161 documents, spanning 456 trials, were analyzed. The majority (126/161) were rated as having information sometimes present (score of >33 to 66%). No SBDs had either no information on any item, or 100% of the information. Items in the patient characteristics component scored poorest (mean component score of 40.4%), while items corresponding to basic trial information were most frequently provided (mean component score of 71%).

**Conclusion:**

The significant omissions in the level of clinical trial information in SBDs provide little to aid clinicians in their decision-making. Clinicians’ preferred source of information is scientific knowledge, but in Canada, access to such information is limited. Consequently, we believe that clinicians are being denied crucial tools for decision-making.

## Introduction

When pharmaceutical companies want to market a new drug, or obtain a new indication for an existing drug in Canada, they are required to submit the entire clinical trial portfolio for that drug to Health Canada. Pivotal trials are regarded as being most important in showing that the product is efficacious and safe in the context in which it will be used. However, Health Canada considers all company-submitted clinical trial data as confidential and will not release it to outside parties, even through an Access to Information request, unless the company submitting the data agrees to its release. [1] This creates a situation where Health Canada possesses information that may be critical to the proper use of a medication but cannot share that information with the people most in need of it – clinicians and patients.

The transparency of Health Canada’s drug review process has been criticized on separate occasions by its Science Advisory Board [2] by the House of Commons Standing Committee on Health [3], and the Auditor General of Canada. [4] The Canadian agency is not alone in this regard: both the European Medicines Agency (EMA) and the United States (US) Food and Drug Administration (FDA) have been criticized in the past for similar shortcomings in transparency. [5] [6] These agencies, however, have met their reproaches with substantial efforts to improve: The FDA already publishes analyses and other materials related to the evaluation of an approved drug on its website, and the EMA’s recently drafted policy on clinical trial transparency (Policy 70) indicates that the agency is moving towards full disclosure of all submitted clinical trial data by 2014 [7].

**Figure 1:**
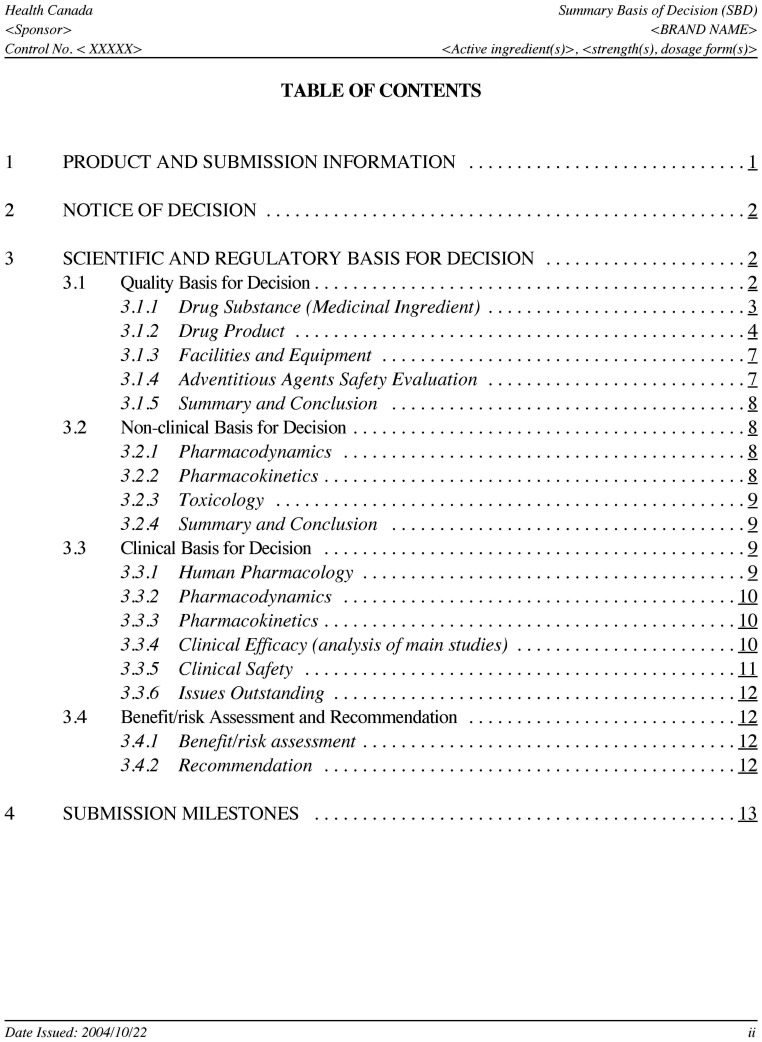
Example of Table of Contents in a Summary Basis of Decision document.

In 2004, Health Canada announced the Summary Basis of Decision (SBD) project. Phase I of this initiative began on January 1, 2005. The SBD is a document issued after a new drug or medical device is approved and explains the scientific and benefit/risk information that was considered prior to approving the product. [8] Technical writers redact the documents based on the reviewers’ report, and revise them upon input and comments from the review team and the sponsoring company. [9] A general template drafted by Health Canada outlines the information to be included in the four major sections of the SBD (Figure 1): 1) Product and Submission Information 2) Notice of Decision 3) Scientific and Regulatory Basis for Decision and 4) Submission Milestones. Of particular interest to healthcare professionals is the third section, which contains a description of the premarket clinical trials examined by Health Canada, and a summary of the final benefit/risk assessment for the product. Health Canada’s position is that, as a result of this initiative, “Canadian healthcare professionals and patients will have more information at their disposal to support informed treatment choices.” [8] SBDs released until the end of August 2012 constituted the first phase of the project [10].

An examination of the strengths and weaknesses of regulatory transparency in one jurisdiction is valuable not only to those in that jurisdiction but also to regulators, clinicians and consumer in other countries. Learning from mistakes and successes can be of significant benefit in understanding how to expand access to necessary and important information in clinical decision-making.

The only published analysis of the contents of the SBDs looked at three pilot documents that were released prior to the launch of Phase I, and found that, compared to the FDA approval package for these drugs, the information contained in the SBD could not alert the reader to the potential problems that would later emerge in Health Canada’s warning letters. [11] In this study, we adopt the perspective of clinicians in analyzing the practical utility of SBDs. Specifically, we ask whether SBDs clearly report information that clinicians would want to know when prescribing a new drug: are the patients in the trials described in enough detail that clinicians would know if they resemble their own patients, and is there enough detail about the trials that they can gain a sufficient understanding of the risks and benefits of the drug? Secondarily, we investigate whether basic clinical trial characteristics are documented.

## Methods

SBDs are available on-line at <http://www.hc-sc.gc.ca/dhp-mps/prodpharma/sbd-smd/drug-med/index-eng.php>. All documents produced between January 1, 2005 and April 30, 2012 were coded. SBDs that did not describe any trials were excluded. Reliability of coding was ensured through duplicate independent coding of a subset of SBDs. RH abstracted information from the first 5 SBDs by alphabetical order according to brand name. JL subsequently did the same and the two sets of data were compared and differences resolved by consensus. JL then did duplicate data abstraction on every 10^th^ document. Consensus was reached on all information extracted. We looked specifically at the Notice of Decision, Clinical Efficacy, Clinical Safety, and Benefit/Risk Assessment and Recommendation sections of each document, as these were the areas where clinical trial information was found. We searched for the following general items for each product: brand and generic name, name of company marketing the product, date of notice of compliance (NOC) and indication(s) for use. Although our aim was to extract information from the pivotal trials described in the SBD, it was not always possible to identify which trials were pivotal due to unclear or ambiguous wording. As such, we extracted information from all trials described in the SBD, unless it was specifically stated that a trial was “supportive”, in which case it was excluded. The following 14 items of clinical trial information were recorded : whether the trial was identified as “pivotal”, the number of pivotal trials per SBD, trial identifiers, trial inclusion requirements, whether the trial was single or multisite, whether the trial was conducted in an inpatient or outpatient setting, the use of placebo or active control, number of patients in each arm, sex distribution in each arm, length of trial, age of patients, results, statistical significance of results, number of withdrawals from each arm of the trial and statistically significant difference, if any, between withdrawal rates.

Each item in a trial was given a score of 2 if it was described completely based on an *a priori* set of definitions (Table 1). If there was some information about the item, but it was incompletely or inaccurately described, it was scored as 1 and if there was no information it was scored as 0. Some trials were observational studies and for these, certain items were expected to be absent (e.g., statistical significance of results). In these cases, items were scored as “not applicable”. For readability, we hereafter refer to both “trials” and “studies” collectively as trials.

**Table 1:** Criteria used to determine presence of item in Summary Basis of Decision documents.

Item	Criteria
Age	Age range and mean or median age of participants in each study.
Gender	Absolute number of male and female participants in each arm of each study or else able to calculate numbers from other data in SBD.
Inpatient/outpatient	Clear statement referring to the setting of each study (inpatient or outpatient). Alternatively, studies in which setting was obvious due to the nature of the indication (i.e. patients receiving treatment for chronic pain would most likely be treated in the outpatient setting).
Trial inclusioncriteria	Specific statement conveying an understanding of who qualified to enter each study.
Treatmentlength	Duration of time that patients were treated with study drug for each study; range of time for the treatment period was acceptable; qualitative descriptions of study length (i.e. time to disease progression) supported by a numerical measure such as the mean.
Results	Description of the primary efficacy endpoint for each study and the number of patients per arm achieving the primary efficacy endpoint or the mean score per arm for each efficacy endpoint. The absolute number of patients per arm achieving the primary endpoint was necessary where absolute number of patients per arm was not disclosed elsewhere.
Statisticalsignificanceof results	Description of the p-value or confidence intervals for the primary efficacy endpoint in each study. At minimum, a clear statement confirming the drug’s non-inferiority or superiority for the primary efficacy endpoint when compared with control arm or the absence of statistically significant primary endpoint results (i.e. Drug X was statistically significantly better than Y for the primary endpoint). For single arm trials, this item was graded as not applicable.
Comparatortherapy	Type of control (i.e. placebo or active) used in each study. If the study was single arm, this item was graded as not applicable.
Withdrawalrate	Description of the absolute number of discontinuations and deaths, for all reasons, in each arm of each study. The withdrawal rate could be described in relative terms (i.e. percentages) if the corresponding number of patients per arm for the study was given elsewhere.
Statisticalsignificance ofwithdrawal rate	Description of the p-value or confidence intervals for the withdrawal rates per arm. At minimum, a clear statement confirming the presence or absence of statistical significance for trial discontinuations per arm was necessary. For single arm trials, this item was graded as not applicable.
Pivotal trialstatus	Clear statement referring to whether each study described in the SBD was pivotal or not.
Number ofpatientsenrolled	Absolute number of patients enrolled in each arm of each study, or the ratio of randomization to each arm, e.g., 1∶1, 1∶2, etc. combined with the total number of patients in the study. If the study was single arm, then the total number of patients enrolled in the study was accepted.
Single ormultisite	Statement for each study describing whether the study was single or multisite.
Study ID	Specific identifier that could be used by the reader to distinguish between different studies described in the SBD.

The unit of analysis was the SBD, as this represents the totality of information for each medication. We totaled the number of points that each SBD received and expressed it as a percentage of the total number of points that were attainable. Therefore, if there were 4 individual trials and there were 12 scoreable items per trial then the maximum number of points would be 96 (4 (trials) ×12 (scoreable items) ×2 (maximum number of points per item)). If the SBD received 72 of these then we reported that it received a score of 75%. We grouped SBDs into categories based on the quantity of information present: information always absent (SBD score of 0%), information usually absent (1 to 33%), information sometimes present (>33 to 66%), information usually present (>66 to 99%) and information always present (100%).

Besides assigning a total score to each SBD, we also identified three component scores, based on the three questions we posed earlier. Items were assigned to these different components based on face validity. For clinicians to be able to know if trial participants resembled their own patients we totaled scores for: age, sex, inpatient or outpatient setting, and inclusion criteria. For clinicians to gain a sufficient understanding of the risks and benefits of the drug we totaled scores for: study length, results, statistical significance of results, placebo or active control, study arm withdrawal rate and statistically significant difference, if any, between withdrawal rates. Finally, basic characteristics were: trial identified as pivotal, number of trials, number of patients per trial arm, single or multisite, and unique trial identifier. SBDs were grouped into the same categories described above for these components as well.

## Results

Excluding four SBDs that did not describe any clinical trials, we analyzed the full population of SBDs available: 161 SBDs covering 456 trials, with a range of 1 to 26 and a mean of 3 trials per SBD (see Table S1 for a full list of the drugs). Overall analysis of the points earned by each SBD is shown in Table 2. Information was neither always absent nor always present in any SBD. The majority of SBDs (126/161) were rated as having information sometimes present, while 30 had information usually present and 5 information usually absent. Examples of SBDs that scored especially poorly as a percentage of the maximum possible score included tositumomab (Bexxar Therapy) (14.3%), ciclesonide (Alvesco) (24.2%), sulesomab (Leukoscan) (27.4%), and abatacept (Orencia) (31.2%). These SBDs failed to describe basic items such as whether trials were controlled or observational, or risk/benefit items such as the outcomes of primary efficacy endpoints. Some SBDs also mentioned trials in passing, without further elaborating upon their characteristics and results. The SBD for the pneumococcal conjugate vaccine (Synflorix), for example, indicated that 11 studies were used to render a decision, but went on to describe only 2. Conversely, the SBDs with the highest percentage of the maximum possible score were alemtuzumab (Mabcampath) (83.3%), denosumab (Prolia) (78.6%), cabazitaxel (Jevtana) (78.6%) and ceftobiprole (Zeftera) (78.6%). The highest scoring SBDs described only one to two trials each whereas the lowest scoring SBDs described between three to 21 trials each (the SBD for tositumomab never disclosed the number of trials reviewed.).

**Table 2:** Scores of components and individual items of Summary Basis of Decision by quintiles.

Component	Individual item	0–20%	>20–40%	>40–60%	>60–80%	>80%	No. of SBDs*analyzed	Mean component scoreas a percent of maximum
**Patient** **characteristics**	Age	109	7	40	1	4	161	40.1
	Gender in each trial arm	129	3	15	0	14	161	
	In/outpatient	64	0	35	1	61	161	
	Eligibility criteria	13	5	22	5	116	161	
**Drug risks** **and benefits**	Length of study	21	5	33	12	90	161	53.2
	Results	1	3	107	10	40	161	
	Mention of statisticalsignificance of results	34	8	43	9	60	154	
	Comparator (placebo or active)	2	1	9	9	140	161	
	Withdrawal rate	70	8	79	2	2	161	
	Mention of statistical significancebetween withdrawal rates	153	0	1	0	0	154	
**Basic trial** **characteristics**	Pivotal status	2	0	34	3	122	161	70.7
	Number of patients	11	6	79	8	57	161	
	Single or multisite	50	1	6	3	101	161	
	Study ID§	54	0	0	1	106	161	
**Overall**		1	14	85	60	1	161	

*Summary basis of decision.

§ Identification number.

Of the three components examined in this study, patient characteristics were the least adequately described. For two items in this component, information was completely absent for the large majority of SBDs: sex per arm (129), and age (108). Eligibility criteria earned the most points in this component, with 114 SBDs containing 100% of the information. The overall patient characteristics score, based on the mean scores on all four related items, was 40.1%.

The second component, drug risks and benefits, fared slightly better (mean component score 53.2%). 135 SBDs fully disclosed the comparator(s) used in trials, and 90 the trial length. The discussion of trial results was less fulsome however, with 110 SBDs having the information only sometimes present. Of the 154 applicable SBDs, the majority (85) had the statistical significance of results always absent to sometimes present. Even less information was provided on the withdrawal rate per arm, with 157 SBDs having the information absent to sometimes present. In 153 of the 154 applicable SBDs, no information was provided on whether withdrawal rates differed significantly between patients in the treatment and control arms.

Basic trial characteristics were most often described in their entirety (mean component score of 71.0). 122 SBDs clearly described whether the trials were pivotal or supplementary, 105 SBDs gave the study name or identifier, 99 the study site and 53 the number of patients per arm.

While the unit of analysis in this study was the entire SBD, an analysis of individual trials provided additional insights (Table 3): the items age, sex per arm, withdrawal rate and statistical significance of differences between withdrawal rates all had a median score of 0 (interquartile range 0, 1). The type of comparator was the only item that attained a median score of 2 (interquartile range 2, 2). Other items that attained a median score of 2 albeit with more dispersed interquartile ranges included study ID, study site, pivotal status, length of study, and eligibility criteria.

**Table 3:** Clarity of information of individual items in all clinical trials.

Component	Individual item	Absent( = 0)	Unclear( = 1)	Present( = 2)	MeanScore	Total number ofclinical trials analyzed
**Patient** **characteristics**	Age	306	140	10	0.18	456
	Gender in each trial arm	381	53	22	0.11	456
	In/outpatient	170	70	216	0.55	456
	Eligibility criteria	49	105	302	0.78	456
**Drug risks** **and benefits**	Length of study	77	122	257	0.70	456
	Results	9	370	77	0.57	456
	Mention of statisticalsignificance of results	120	165	152	0.54	437
	Comparator (placebo or active)	13	56	387	0.91	456
	Withdrawal rate	248	200	8	0.24	456
	Mention of statistical significancebetween withdrawal rates	435	2	0	0.00	437
**Basic trial** **characteristics**	Pivotal status	6	153	297	0.82	456
	Number of patients	57	268	131	0.58	456
	Single or multisite	214	1	241	0.53	456
	Study ID*	183	1	272	0.60	456

*Identification number.

## Discussion

The stated aims of the SBD initiative are to improve transparency in the drug review process, and to provide physicians and the public with access to unbiased information regarding authorized products. [8] While the initiative is a laudable departure from a complete lack of data, our findings point to significant room for improvement: Overall, clinical trial information in SBDs is presented in a haphazard manner, with no apparent method to its presentation. The majority of SBDs (126 of 161) obtained overall scores of less than or equal to 66% of the total number of available points, meaning that at least one-third of the potential information about patient trial characteristics and the benefits and risks of tested treatments is missing. While basic details of clinical trials were more frequently described, any omissions or ambiguities in this component were especially troubling given the straightforward nature of the information that needed to be conveyed. In its Phase I form, the SBD offered only a very modest quantity and quality of information to aid in clinical decision-making.

Physicians have good reason to regard newly approved drugs with caution: between 1990 and 2009, 4.2% of the drugs approved by Health Canada were subsequently withdrawn due to safety issues. [12] Overall, new drugs have almost a 25% chance of acquiring a serious safety warning or being removed from the market and for drugs approved through the priority review process (180 days compared to 300 days for the standard process) that number climbs to 34%. [13] Efforts in the US to meet deadlines for reviews have also been associated with an increased likelihood of drug withdrawals for safety reasons [14].

Safety issues discovered through post-market surveillance can help clarify the benefit-to-harm ratio of drugs but knowledge of these problems is not available early on in the lifecycle. Additionally, access to more complete information from the premarket trials would enable clinicians to better contextualize post-market safety signals by asking, for example, whether safety problems were entirely new discoveries or further evidence of a signal that was evident in the premarket trials.

Physicians prefer to use scientific knowledge in making prescribing decisions, [15] but when drugs first appear on the market there is little peer-reviewed published information. [16], [17] Reliance on the eventual publication of the clinical trials is not enough: in the US, almost one-quarter of the pivotal trials for FDA approved drugs remained unpublished >5 y after approval. [16]. Moreover, the publication of those studies with positive results (publication bias) and the reporting of outcomes with the most impressionable findings (outcome reporting bias) can significantly alter the apparent efficacy of drugs, [18] misleading clinicians [19], [20].

Additionally, there are often marked discrepancies between the results of trials submitted as clinical study reports (CSRs) to regulatory agencies, and the results that appear in publications. While this observation may be partly due to the restrictions imposed by journals, the bias has been consistently in favor of the company funding the research: Vedula et al, for instance, found that publications did not accurately reflect the efficacy results reported in internal company documents [21] and Wieseler and colleagues showed that the clinical study reports (CSRs) provided considerably more information on harms than did publicly available sources including journal publications and registry reports [22].

It is also important to consider that new drugs are typically first approved on the basis of clinical trials, which have stringent eligibility criteria, and a relatively homogeneous patient population. [23] The extrapolation of trial results to the diversity of patients in a physician’s practice is a detail-driven process, and without in-depth knowledge of the characteristics of trial participants, extrapolation becomes much more difficult. Our analysis indicates that Phase I SBDs cannot provide the depth of information that physicians need to translate the results of clinical trials in treatment for the patients that they see in their offices.

Although basic trial information was most frequently described, nearly one quarter (24.2%) of the SBDs failed to indicate whether one or more of the trials that were described were pivotal. It is difficult to understand why this information should be so frequently absent in a decision summary document, since pivotal trials are key to making decisions about approval. Another item in this category, the study identifier, was even more rarely found (54 SBDs had no identification for any of their associated trials). Study identifiers are useful for determining whether clinical trials have been published, and for checking whether the trial has been registered on clinical trial registries such as ClinicalTrials.gov. Physicians’ access to this type of information would enhance their appraisal of the quality of evidence available for a newly approved drug.

Health Canada’s own evaluation of Phase I SBDs, based on the 93 SBDs published up to September 2008, complemented our analysis by revealing that a little over half of respondents to a workbook published on the SBD website found SBDs “useful in helping them make informed treatment choices (for themselves or their patients)”. [24] However, the report did not disclose either the percent or the absolute number of respondents who were clinicians. The evaluation also acknowledged the varying quality of information in the documents, but attributed it to causes such as the quality of review report, skill of the technical writer and nature of the drug. In Phase II, SBDs have been restyled into a web-based, question-and-answer format with inter-document links to improve navigation. Asked whether the content of the SBDs will be the same as in Phase I, Health Canada responded that the new SBDs would have more information on risk/benefit analyses. [9] It is unclear whether this means that there will also be more in-depth information about the results and characteristics of the clinical trials submitted by the companies.

The SBD initiative drew inspiration from the European Public Assessment Report (EPAR) started in 1995 by the European Medicines Evaluation Agency (now EMA). [8] But the quality of information contained in EPARs has also been criticized in the past. [25], [26] In their analysis of the quality of information in EPARs for psychiatric drugs, Barbui and colleagues described an erratic reporting style and revealed that under 50% of the 70 trials described in the EPARs disclosed information about the number of patients allocated to each arm, the number withdrawn in each arm, or the number included in the analysis of the primary outcome (with effect size and precision). [25] These findings are consistent with our analysis of the SBDs, and as Barbui et al point out, such irregular and unreliable styles of reporting render it impossible to use these documents for analyses of treatment effect. We agree with the authors that a minimum first step towards improving information quality in these documents would be the adoption of a table to systematically organize trial information and results. Tabular presentations, however, would not obviate the need for more commitment on the part of Health Canada, as in the EMA, to disclose clinical trial information pertaining to newly approved drugs. In this regard, disclosure of the complete CSR would provide access to additional important data.

Calls for data transparency are steadily accruing from stakeholders groups across the globe, [27] and there is now a realization, even amongst some pharmaceutical companies, [28] that the era of data secrecy may be nearing its end. Viewed in this international context, and sandwiched between the considerably more transparent policies of the FDA and the EMA, Health Canada’s initiative appears conspicuously opaque.

There are several limitations to this paper. First and foremost, our assumption about the type and quantity of information that physicians need in order to make informed clinical decisions has only face validity. There are many factors that influence how doctors use new drugs, [29], [30] but doctors seem to seek information about the products’ safety and effectiveness from all sources. [31] Therefore, we feel that our focus on these areas in the SBDs is justified. Second, we’ve accorded every item in this study equal weight, though clinicians might not necessarily value all facets of clinical trial information equally (e.g., the statistical significance of a result might be more important to a physician than the numerical result itself). It is quite likely that doctors stratify the amount of information they desire based on the perceived risk of drugs and that consultants and general practitioners behave differently. [29] It should also be noted that other information missing from the SBD could also have significant clinical importance. It was only by examining the full CSRs that the authors of a recent Cochrane review of neuraminidase inhibitors were able to determine that the increased incidence of gastrointestinal side effects in the group taking the placebo may have been due to ingredients in the placebo. [32] There is an increasing recognition of the value of regulatory documents such as CSRs in evidence synthesis and review studies. [33], [34] SBDs also have the potential to play a greater role in bolstering the findings of systematic reviews and evidence synthesis documents specific to the Canadian clinical context. Finally, we understand that even if the amount of information in the SBD was significantly expanded, that clinicians may not consult these documents for guidance in day-to-day prescribing decisions. However, even if they do not, the information in them would be of significant value to those who formulate clinical practice guidelines and assemble drug formularies.

Health Canada claims that its SBD project has two goals: 1) to improve the transparency of the review process, and 2) to provide Canadians with unbiased information. Part of the latter goal is to help healthcare professionals make unbiased decisions. To date the evidence suggests that the SBDs are ineffective in reaching that goal. There are minimal legal barriers preventing Health Canada from disclosing more data [1] and without that information we believe clinicians are being denied crucial tools for decision-making.

## Supporting Information

Table S1 List of drugs with Summary Basis of Decision.
(XLS)
